# Smart and bioactive packaging systems from anthocyanins and zinc oxide nanoparticles for quality monitoring and shelf-life extension of Nile perch (*Lates niloticus)*

**DOI:** 10.1038/s41598-026-58478-y

**Published:** 2026-06-22

**Authors:** Ahmed A. Tayel, Norhan I. Gomaa, Osama M. Abonama, Ayman Y. Allam

**Affiliations:** 1 Department of Fish Processing and Biotechnology, Faculty of Aquatic and Fisheries Sciences, Kafrelsheikh University, Kafrelsheikh, 33516 Egypt; 2 Department of Industrial Biotechnology, Faculty of Biotechnology, University of Sadat City, El-Sadat City, 32897 Egypt; 3 Department of Food Science and Technology, Faculty of Agriculture, Menoufia University, Shibin El Kom, 32511 Egypt

**Keywords:** Anthocyanin, *Hibiscus sabdariffa*, Smart indicator, Green synthesis, Zinc oxide nanoparticles, Chitosan nanoparticles, Fish quality monitoring, Biochemistry, Biotechnology, Chemistry, Environmental sciences, Materials science, Nanoscience and technology

## Abstract

Anthocyanins possess high potentiality as natural pH-sensitive pigments, enableing their usages as safe alternatives for monitoring food quality. This study targeted the development of smart, active, and bioactive dipping solutions (SCS), comprising anthocyanin-rich extract from *Hibiscus sabdariffa* (HE) with green-synthesized zinc oxide nanoparticles (ZnONPs) stabilized in chitosan nanoparticles (ChNPs). The HE displayed distinct color transitions under different pH conditions, e.g. red to pink in acidic, violet in neutral, and green to yellow in alkaline media, signifying its potential as a freshness indicator. Green-synthesized ZnONPs exhibited smaller particle sizes (6.42–15.92 nm) compared to ZnONPs prepared without HE (26.92–41.24 nm). XRD confirmed ZnONP crystallinity, while UV–Vis absorption at 377 nm verified nanoparticle formation. Zeta potential values indicated stability, with ZnONPs (− 28.73 mV), and ChNPs (+ 36.4 mV). The SCS demonstrated strong antioxidant activity (89.29% DPPH scavenging) and antibacterial effects against *Escherichia coli* and *Staphylococcus aureus*. FTIR confirmed successful component interactions, and SEM analysis verified nanocomposite formation. Application of SCS on Nile perch fillets stored at 4 °C effectively delayed spoilage, extending shelf life by up to six days. Moreover, the color transition from red to green during storage provided a visual signal of quality decline. These findings highlight the potential of anthocyanin-based nanocomposite systems with ZnONPs and ChNPs and as eco-friendly smart packaging solutions for real-time fish quality monitoring and preservation.

## Introduction

Fish and fishery products are widely recognized as nutrient-dense foods, providing high-quality proteins, polyunsaturated fatty acids, minerals, and essential micronutrients. However, immediately after capture, fish muscle is highly susceptible to biochemical and microbial deterioration. Proteins and lipids undergo degradation and oxidation under the influence of temperature, humidity, and spoilage bacteria during storage, transportation, and retail, ultimately compromising quality and safety^1^. During storage, fish pH initially decreases due to glycogen breakdown into lactic acid, but subsequently rises as proteins decompose, generating alkaline by-products such as biogenic amines, trimethylamine, dimethylamine, and ammonia^2^. The accumulation of these volatile basic nitrogen compounds (TVB-N) correlates with microbial growth and spoilage progression^3,4^. Thus, pH and TVB-N are widely used as quality indicators in fish freshness evaluation. Anthocyanins, natural flavonoid polyphenols, have gained increasing attention as pH-sensitive, non-toxic pigments. Their color varies with pH: red in acidic conditions, purple at neutral pH, and blue to green in alkaline media, due to structural transformations of their ionic forms^5^. Although anthocyanins are sensitive to pH, light, and temperature^6^,they represent an attractive alternative to synthetic colorants, which are associated with health risks including allergies, behavioral disorders, and certain cancers^7^. Consequently, anthocyanins have emerged as promising candidates for developing eco-friendly, smart food quality indicators.

Chitosan, a biodegradable and non-toxic biopolymer, has been extensively investigated for applications in food packaging^8^. It possesses antimicrobial properties and can be combined with anthocyanins to create active and intelligent packaging systems^9^. Nevertheless, native chitosan films exhibit drawbacks such as low mechanical stability, weak tensile strength, and high permeability, which limit their application. To overcome these limitations, chitosan nanocomposites have been developed. Chitosan nanoparticles (ChNPs), in particular, are attractive due to their high surface area, strong adsorption capacity, and ability to enhance encapsulation and stability of bioactive compounds^10^. Several fabrication methods, including emulsion cross-linking^11^, ionic gelation^12^, and spray drying^13^, have been explored to produce ChNPs.

*Hibiscus sabdariffa* (*H. sabdariffa*), commonly known as roselle, karkadeh, zobo, or sour tea, is traditionally consumed worldwide as a functional beverage. Its calyces are rich in anthocyanins, flavonoids, hibiscus acids, and ascorbic acid, making them valuable natural antioxidants and colorants^14,15^. The predominant anthocyanins in *H. sabdariffa* include cyanidin-3-sambubioside, delphinidin-3-sambubioside, delphinidin-3-glucoside, and cyanidin-3-glucoside, which contribute to its deep red coloration. Beyond their coloring properties, *H. sabdariffa* bioactives have been reported to exert antihypertensive^16,17^, hepatoprotective^18^, cardioprotective^19^, and antidiabetic effects, highlighting its potential as a multifunctional natural additive.

The fabrication of nanomaterials (e.g. metals, metal oxides, polysaccharides, biopolymers, libids, peptides and their composites) attract great interests due to their extraordinary properities and bioactivities^20^. The synthesis of such nanomaterials could involve chemical, phesical or biological approaches, with many advateges of biological (green) synthesis methods that include feasibility, safety and cost effectiveness^20–23^.

Zinc oxide nanoparticles (ZnONPs) have also attracted considerable interest due to their unique optical, antimicrobial, and antioxidant properties, as well as their diverse biomedical applications, including antifungal, anticancer, wound healing, antiviral, and larvicidal activities^20–22^. Unlike many other metal oxide nanoparticles, ZnONPs are generally recognized as safe (GRAS) by the U.S. FDA, offering advantages in terms of biodegradability and minimal toxicity^23^. Green synthesis of ZnONPs using plant extracts is an environmentally friendly and cost-effective approach that enhances nanoparticle stability and biocompatibility while avoiding toxic reagents typically used in chemical or physical methods^22,24^.

Regarding these considerations, this study aimed to develop a novel smart, functional, and bioactive dipping solution (SCS) via incorporating *H. sabdariffa* extract (HE) and green-synthesized ZnONPs encapsulated within ChNPs. The proposed system was designed to both extend the shelf life of Nile perch (*Lates niloticus*) during refrigerated storage at 4 °C and serve as a freshness indicator by detecting pH-associated spoilage changes.

## Materials and methods

### Materials

Dried *Hibiscus sabdariffa* flowers were obtained from the Agricultural Research Centre, Giza, Egypt. Fresh Nile perch (*Lates niloticus*) samples were sourced from the aquaculture research farm at Kafrelsheikh University, Egypt. Ethanol (96%), low molecular weight chitosan powder (degree of deacetylation 90%), sodium tripolyphosphate (STPP), absolute acetic acid, zinc acetate anhydrous, and sodium hydroxide (NaOH, 0.1 M) were purchased from Sigma-Aldrich (Germany). Distilled water was used throughout all experiments. The plant materials were identified by the academic botanist, Dr. Khaled E. Mazrou, at University of Sadat City, Egypt. The plant materials’ handling was complied with the IUCN Policy Statement on Research Involving Species at Risk of Extinction and the Convention on the Trade in Endangered Species of Wild Fauna and Flora.

**Fig. 1:**
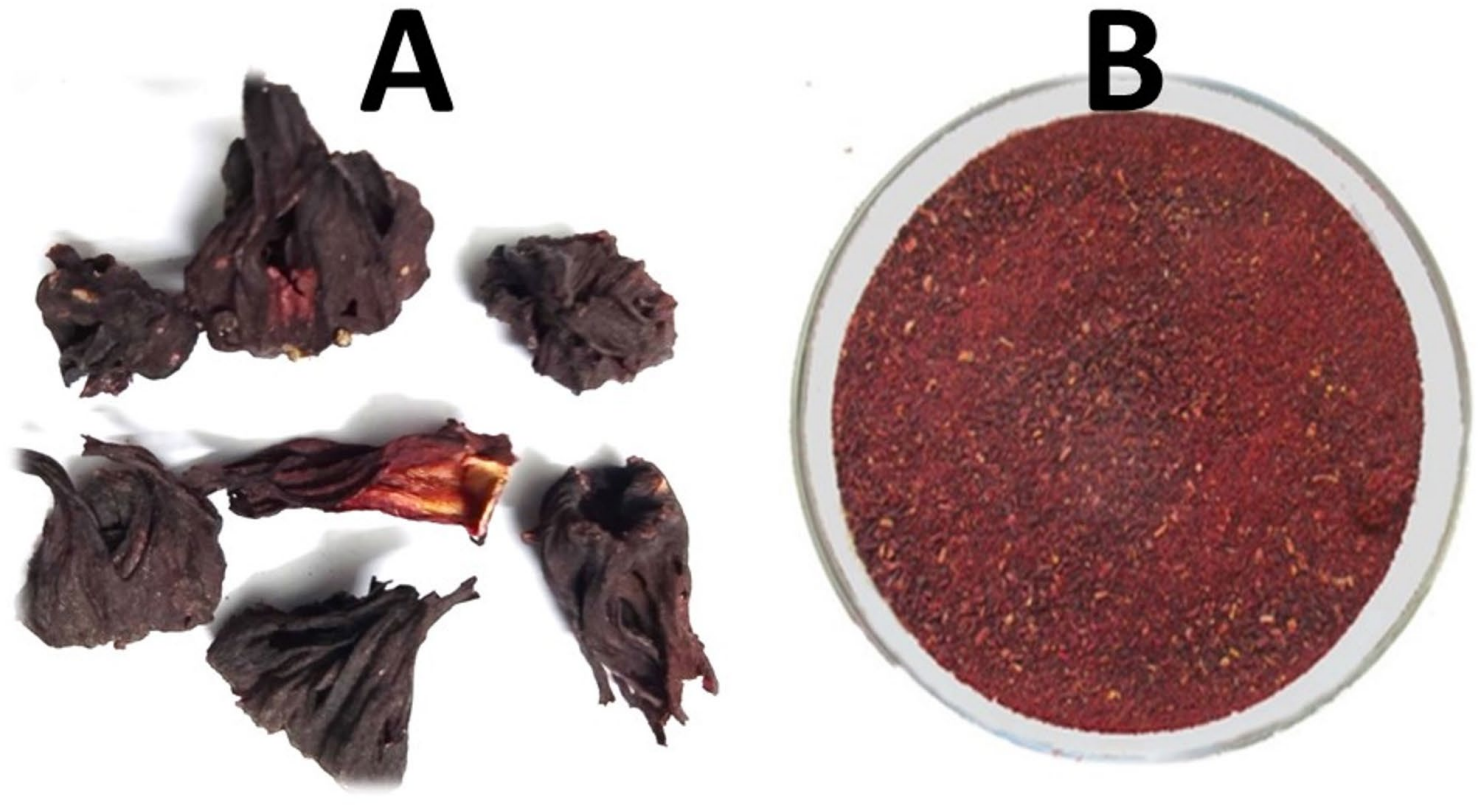
Dried flowers (**A**) and powder (**B**) of *Hibiscus sabdariffa*, used in this study as a natural source of anthocyanins for smart colorimetric indicator preparation.

### Methods

#### Preparation of *H. sabdariffa* extract (HE)

Ten grams of dried *H. sabdariffa* flowers were finely ground (Fig. 1) and soaked in 96% ethanol for 12 h, following a previously reported method with minor modification such as removing heat^20,21,23^. The mixture was stirred at 800 rpm for 2 h using a magnetic stirrer, followed by filtration through muslin cloth. The filtrate was concentrated at 45 °C until dryness to obtain the anthocyanin-rich extract (HE).

#### Green synthesis of zinc oxide nanoparticles (ZnONPs)

One gram of zinc acetate anhydrous was dissolved in 100 mL of distilled water until complete dissolution, resulting in a whitish solution. Subsequently, 1 g of HE was added under continuous stirring. The pH was adjusted to 12 by the gradual addition of 0.1 M NaOH until the solution turned yellow, indicating nanoparticle formation. The resulting precipitate was centrifuged at 800 rpm for 20 min, washed repeatedly with distilled water, and dried at 50 °C for 1 h^20^. The dried material was then calcined at 350 °C for 2 h to obtain ZnONPs. For comparison, a control sample was prepared without the addition of HE (W-ZnONPs). The resulting nanopowders were subjected to physicochemical characterization.

#### Preparation of smart dipping solution (SCS)

One gram of chitosan powder was dissolved in 100 mL of 1% (v/v) acetic acid under stirring at 50 °C for 2 h to prepare a chitosan solution (CHS). Subsequently, 0.5 g of HE and 0.5 g of ZnONPs were added to 50 mL of CHS while stirring. In parallel, 25 mL of STPP solution was added dropwise to 25 mL of CHS to induce ionic gelation. The resulting mixture was blended with HE and ZnONPs to form the final smart dipping solution (SCS), which was then subjected to structural and functional characterization^20,24^.

#### Application of SCS on fish samples

Fresh Nile perch fillets were thoroughly washed, descaled, and deboned. The flesh was cut into cubes weighing approximately 39 ± 2 g each. The cubes were immersed in 10 mL of the smart dipping solution (SCS) for 3 min, ensuring complete coverage of all surfaces. Treated samples were stored at 4 °C for 7 days. Untreated fish cubes served as controls. All samples were periodically examined to evaluate spoilage progression and shelf-life extension^27^. The spoilage-related indicators, e.g. samples pH and TVB-N “total volatile basic nitrogen” were measured during the fillets samples storage period^2,53^. For pH determination, 10 g of fish fillet were aseptically attained. After homogenising with 90 mL of distilled water for 2.0 min, the pH meter (AD-1040, Adwa, Szeged, Hungary) was applied at room temperature. The assessment of TVB-N followed the official hydrodistillation technique, involving using alkaline solution then titrating versus ammonia yield. Firstly, homogenized flesh (10 g) was intermixed with trichloroacetic acid (7.5%; 20 mL) and sieved through filter membrane. The filtered yield was hydrodistillated (VELP UDK-6; Milan, Italy), alongside mixing with NaOH (10%; 3 mL). Concentrated vapor was assembled in 4% boric acid, accompanied with 1 mL of blended index (1: 2 ratio of methylene blue: methyl red). The developed alkaline mix was titrated with H_2_SO_4_ (0.025 N).

#### Characterization of ZnONPs, HE, and SCS

##### pH sensitivity of HE

The pH responsiveness of *H. sabdariffa *extract (HE) was assessed using 1 M acetic acid (for acidic conditions) and 1 M NaOH (for alkaline conditions). Color transitions were recorded across different pH values to evaluate suitability as a freshness indicator^26^.

##### Transmission electron microscopy (TEM)

TEM “Leica-Leo 0430; Cambridge, UK” was performed to determine the morphology and particle size of ZnONPs synthesized with HE and without HE (W-ZnONPs). Samples were dispersed in distilled water, sonicated for 5 min, and drop-cast onto carbon-coated copper grids, vacuum-dehydrated for ∼35 min, before subjecting to 20 kV operated TEM^4^.

##### X-ray diffraction (XRD)

XRD analysis was conducted to determine the crystalline structure and phase purity of ZnONPs. Diffraction patterns were recorded using an X-ray diffractometer “XRD-6000; Shimadzu Co., Kyoto, Japan”, and peak positions were compared to standard ZnO reference patterns^4^.

##### UV–visible absorption spectroscopy (UV–Vis)

The optical properties of ZnONPs were characterized using a UV–Vis spectrophotometer (Eppendorf Biospectrometer, Hamburg, Germany). For analysis, 10 mg of ZnONPs was resuspended in 15 mL of distilled water and sonicated for 10 min. The absorption spectrum was scanned in the range of 300–700 nm to detect characteristic peaks confirming nanoparticle formation^20^.

##### Zeta potential measurement

The surface charge and colloidal stability of nanoparticles were assessed by measuring the zeta potential of ZnONPs, W-ZnONPs, and ChNPs. Measurements were carried out with zetasizer “Malvern, Worcestershire, UK” at room temperature in distilled water as the dispersing medium^7^. Zeta potentiality (ζ) were recorded between + 150 and − 150 mV at 30 °C.

##### Antioxidant activity (DPPH assay)

The antioxidant activity of SCS was evaluated using the DPPH radical scavenging assay, following the method of **Sánchez-Moreno**^25^, with slight modifications. Briefly, serial dilutions of the sample were prepared in methanol. A 0.135 mM DPPH solution was mixed in equal volumes with each dilution, incubated in the dark for 30 min at room temperature, and the absorbance was measured at 517 nm. Ascorbic acid was used as the positive control. Radical scavenging activity was calculated as:

1 $$\rm{\%\: Scavenging\: Activity}=\frac{A_{control}-A_{sample}}{A_{control}}\times100$$

*Where A*_***control***_ is the absorbance of the DPPH solution without a sample, and *A*_*sample*_ is the absorbance with a sample.

##### Antibacterial activity assay

The antibacterial activity of SCS was evaluated using the agar well diffusion method against *Escherichia coli* (ATCC 10536, Gram-negative) and *Staphylococcus aureus*(ATCC 6538, Gram-positive). Mueller–Hinton agar plates were inoculated with 100 µL of standardized bacterial suspensions (10⁶ CFU/mL). Wells of 9 mm diameter were aseptically punched into the agar and filled with 100 µL of SCS. Plates were incubated at 37 °C for 24 h, after which the zones of inhibition were measured in millimeters^23^.

##### Fourier transform infrared (FT-IR) spectroscopy

FT-IR “Thermo Fisher, Nicolete IS10, Waltham, MA” spectroscopy was employed to characterize the functional groups and chemical interactions within the formulations. FT-IR spectra of ChNPs + HE, ZnONPs, and SCS were recorded in the range of 4000–500 cm⁻¹ using a spectrometer^4^. Samples were individually amalgamated with 1% Potassium Bromide, which acted as the carrier with full transmittance, and were inspected to detect the biochemical interactions after molecules merging. The wavenumber range of 4000 cm^− 1^ to 450 cm^− 1^ was operated.

##### Scanning electron microscopy (SEM)

SEM “IT100, JEOL, Tokyo, Japan” was used to investigate the surface morphology and microstructural features of the SCS. The samples were sonicated, mounted onto self-adhesive carbon discs, coated with palladium/gold prior to imaging, and surface topography was examined under varying magnifications to assess structural uniformity and nanoparticle distribution at 10 kV operating acceleration^4,22^.

### Ethical declaration

The study was approved by the “Institutional Aquatic Animal Care and Use Committee” at Kafrelsheikh University, and performed in accordance with the guidelines and regulations set by Agriculture Research Center, Egypt (Approval No. ARC/CLAR/89/24), and in accordance with EU (Directive 2010/63) legislation on the protection of animals used for scientific purposes.

The study experiments also adhered to the regulations and guidelines of ARRIVE (https://arriveguidelines.org/).

## Results and discussion

### pH sensitivity test for HE

**Fig. 2:**
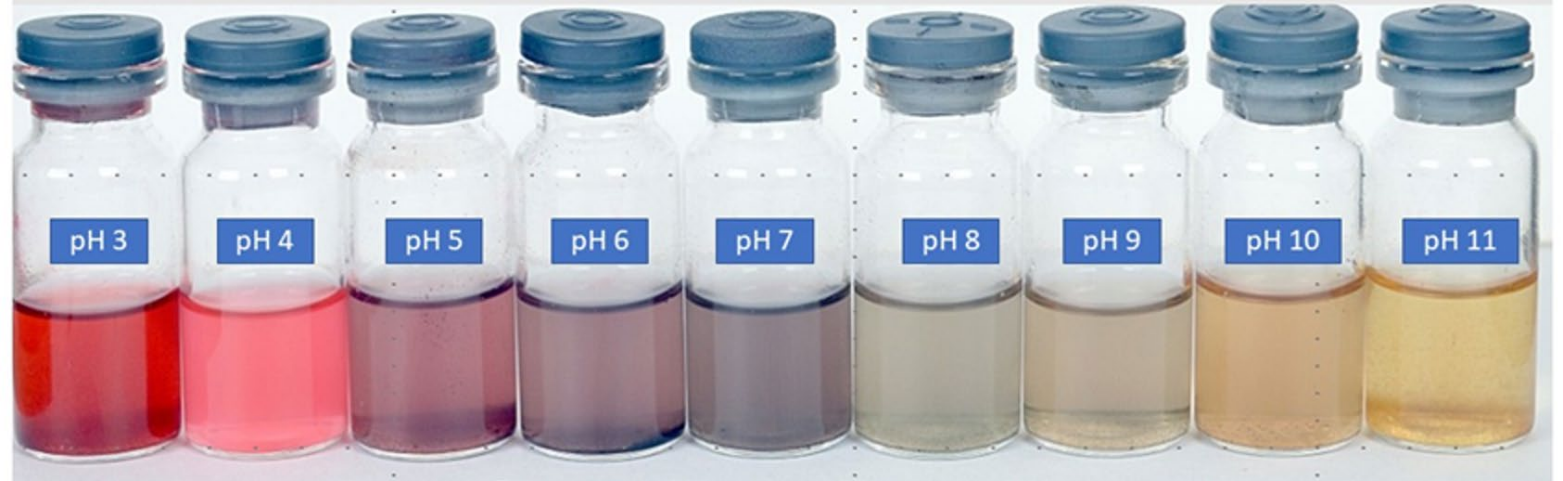
*Color-changing sequence of* H. sabdariffa *extract (HE) under different pH conditions (left to right).*

pH plays a critical role in the color variation of anthocyanin-rich extracts. As illustrated in Fig. 2, the color of HE was red at pH 3, shifted to pink at pH 4, and changed to purple between pH 5 and 7. At alkaline conditions (pH 8–10), the extract exhibited colors ranging from pale green to yellow. These variations are attributed to structural transformations of anthocyanins, such as flavylium cation, quinoidal base, and chalcone forms, which dominate at different pH values^26^. Such reversible color transitions qualify anthocyanins as effective colorimetric indicators for monitoring food freshness by detecting pH changes caused by microbial metabolites during spoilage^27^.

### X-ray diffraction (XRD)

**Fig. 3:**
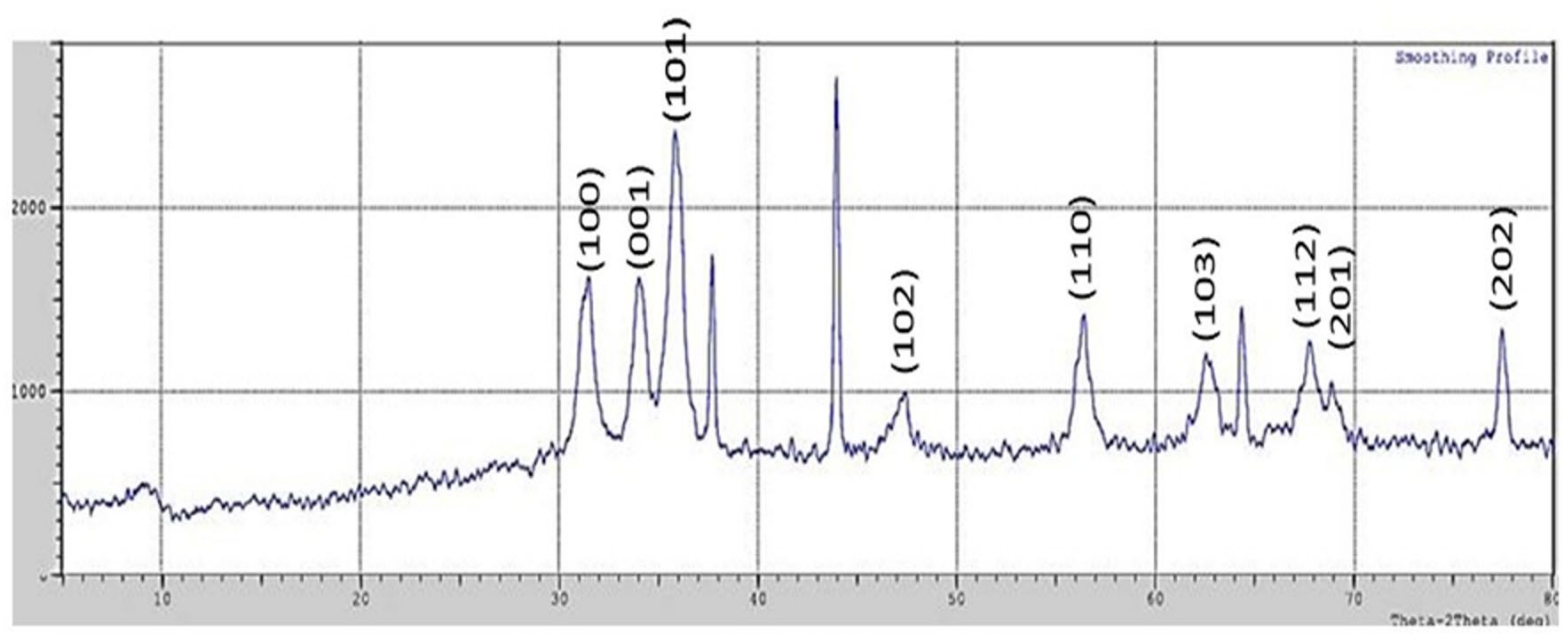
*XRD diffraction pattern of ZnONPs synthesized using* H. sabdariffa *extract confirming hexagonal wurtzite structure.*

The crystalline structure of the synthesized ZnONPs was confirmed using XRD analysis (Fig. 3). The diffraction pattern exhibited characteristic peaks at 2θ values of 31.52°, 34.04°, 35.83°, 47.43°, 56.42°, 62.65°, 68.00°, and 77.50°, corresponding to the crystal planes (100), (002), (101), (102), (110), (103), (112), and (202), respectively. These reflections are in good agreement with the standard hexagonal wurtzite structure of ZnO as indexed by the Joint Committee on Powder Diffraction Standards (JCPDS card no. 36–1451). The most intense peaks at 31.52°, 34.04°, and 35.83° indicate high crystallinity of the prepared ZnONPs^24^.

The Scherrer’s calculation enabled determination of crystalline nature of the ZnONPs, with average particle size of 33.82 nm. The absence of additional impurity peaks suggests the successful formation of pure ZnONPs without detectable secondary phases. However, minor broadening of peaks was observed, which can be attributed to the nanoscale crystallite size and the possible presence of surface strain induced during green synthesis. This is consistent with previous studies where phytochemicals in *H. sabdariffa *extracts acted as capping agents, reducing particle size and influencing crystal growth^36,39,40^.

### UV–visible spectroscopy (UV–Vis)

**Fig. 4:**
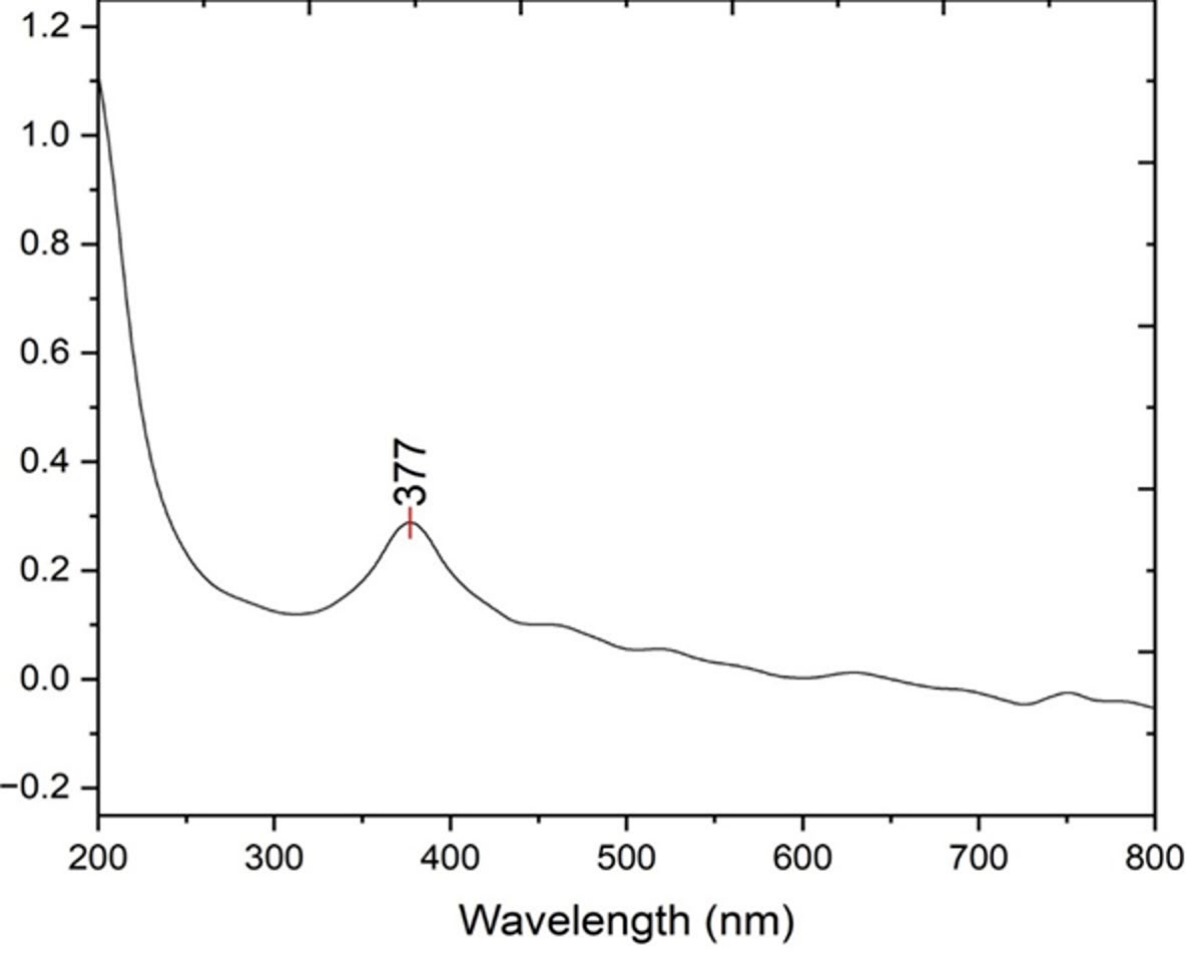
*UV–Vis absorption spectrum of ZnONPs synthesized using* H. sabdariffa *extract*,* showing a characteristic peak at 377 nm.*

The optical properties of the synthesized ZnONPs were investigated using UV–Vis spectroscopy. As presented in Fig. 4, a distinct absorption peak was observed at 377 nm, which is characteristic of the intrinsic band gap absorption of ZnO nanoparticles. No additional peaks were detected, confirming the high purity of the synthesized nanoparticles^41^. This result aligns with earlier reports, which indicated that ZnONPs typically exhibit strong absorption bands within the range of 340–380 nm due to electronic transitions from the valence band to the conduction band^42,43^. The sharpness and intensity of the peak further suggest good crystallinity of the ZnONPs, supporting the findings from XRD analysis.

### Transmission electron microscopy (TEM)

**Fig. 5:**
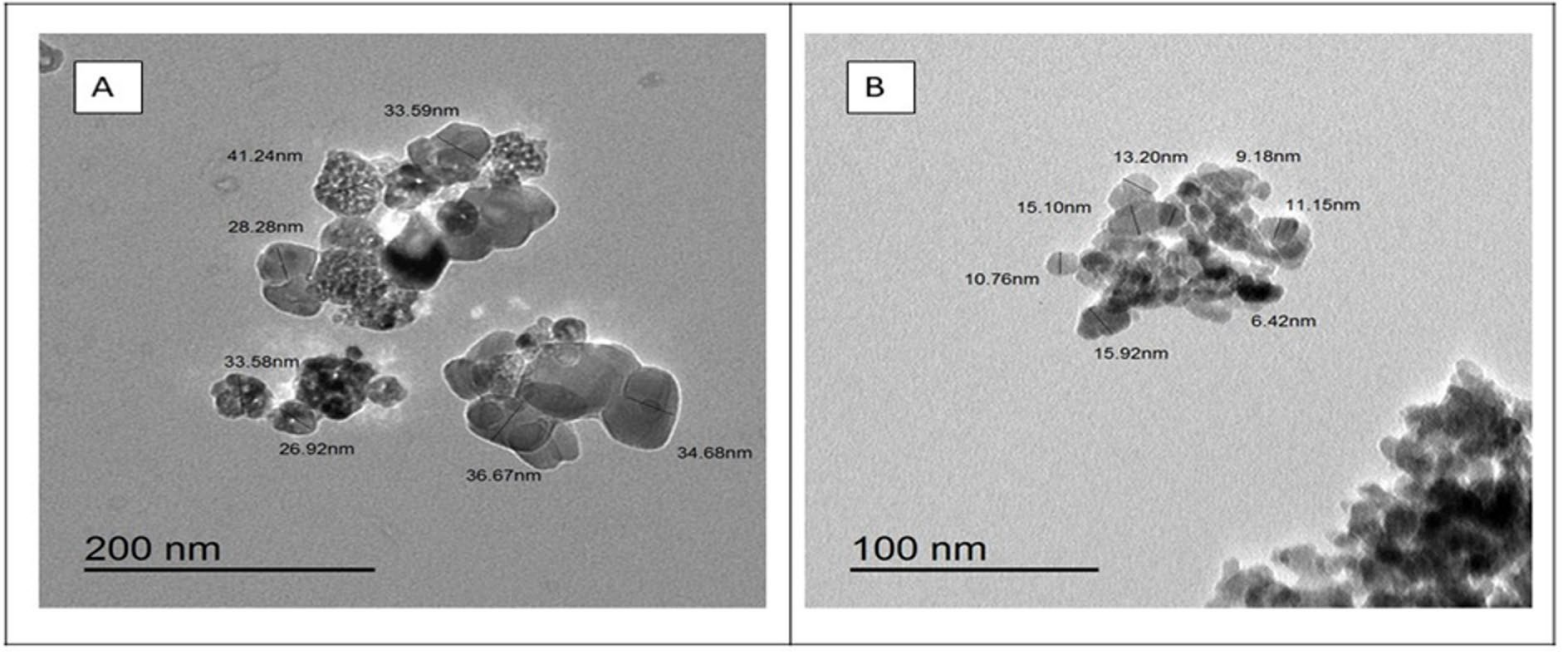
(**A**) TEM micrograph of ZnONPs synthesized using *H. sabdariffa* extract (HE). (**B**) TEM micrograph of W-ZnONPs synthesized without HE.

TEM analysis revealed distinct differences in nanoparticle morphology and size distribution between green-synthesized ZnONPs and chemically synthesized W-ZnONPs. As shown in Fig. 5A, ZnONPs synthesized using HE exhibited relatively uniform spherical to quasi-spherical shapes, with particle sizes ranging from 6.42 to 15.92 nm. In contrast, Fig. 5B shows that W-ZnONPs displayed more irregular shapes and significantly larger particle sizes ranging from 26.92 to 41.24 nm. Notably, both samples exhibited some degree of agglomeration, which is commonly observed in nanoparticles synthesized in aqueous media due to their high surface energy^28^.

Transmission electron microscopy (TEM) was employed to investigate the morphology and particle size distribution of the synthesized zinc oxide nanoparticles (ZnONPs) using *Hibiscus sabdariffa* extract (HE) compared with chemically synthesized ZnONPs without hibiscus extract (W-ZnONPs). As shown in Fig. 3A, the ZnONPs synthesized via the green approach exhibited predominantly spherical to quasi-spherical particles with relatively uniform morphology and sizes ranging between 6.42 and 15.92 nm. In contrast, W-ZnONPs (Fig. 5B) displayed a more heterogeneous morphology, with particle sizes varying widely from 26.92 to 41.24 nm. The observed reduction in particle size and improved uniformity of ZnONPs synthesized using hibiscus extract clearly indicates the role of bioactive phytochemicals in modulating nucleation and growth during nanoparticle formation. The smaller size of ZnONPs in the HE-mediated synthesis can be attributed to the reducing and capping actions of anthocyanins, flavonoids, and phenolic acids present in *H. sabdariffa* extract, which provide electron-rich functional groups that stabilize the nanoparticle surface and prevent excessive agglomeration. These biomolecules act as natural chelating agents, controlling crystal growth and leading to nanoparticles of smaller size with narrower size distribution^26,29^. In contrast, the absence of these phytoconstituents in W-ZnONPs allowed uncontrolled crystal growth, leading to larger and less homogeneous particles. The slight agglomeration observed in both ZnONPs and W-ZnONPs is consistent with reports from aqueous-phase nanoparticle synthesis, where the high surface energy of nanosized particles promotes aggregation^30^. However, the degree of agglomeration in HE-mediated ZnONPs was less pronounced, suggesting that the phytochemicals in hibiscus extract not only controlled size but also imparted partial steric stabilization. Similar stabilization effects have been reported in ZnONPs synthesized using *Moringa oleifera* and *Camellia sinensis*extracts, which reduced particle agglomeration through polyphenol-mediated capping^31,32^. The formation of nanoparticles within the 6–16 nm range is particularly important, as smaller ZnONPs are associated with higher surface-to-volume ratios, which enhance their antioxidant and antibacterial activities. Previous studies demonstrated that ZnONPs below 20 nm exhibit significantly higher generation of reactive oxygen species (ROS) and stronger bactericidal activity compared to larger particles^33,34^. Thus, the particle size distribution observed in the HE-mediated ZnONPs may contribute to the enhanced functional properties of the smart composite system (SCS) developed in this study. Furthermore, the observed uniformity in nanoparticle morphology is critical for their stability and consistent performance when incorporated into nanocomposites. Irregular or highly polydisperse nanoparticles often lead to poor dispersion and heterogeneous functional activity in applied systems^35^. The controlled synthesis mediated by hibiscus extract therefore provides an advantage for food applications, where reproducibility and safety are key considerations. Overall, the TEM results demonstrate that *H. sabdariffa* extract acts as an efficient biotemplate for controlling ZnONPs synthesis, yielding smaller, more uniform, and less aggregated nanoparticles compared to conventional chemical synthesis. These characteristics enhance the functional potential of ZnONPs in active food packaging and smart quality monitoring systems. The findings corroborate earlier studies that reported the superiority of plant-mediated ZnONPs synthesis in producing nanoparticles with desirable size and morphology for biomedical and food applications^36,37^. The reduction in particle size and improved homogeneity in the presence of HE highlight the role of its phytoconstituents (mainly phenolics and flavonoids) as reducing and stabilizing agents during green synthesis. These findings are consistent with earlier studies, which demonstrated that plant-derived bioactives facilitate nanoparticle nucleation and prevent uncontrolled growth, thereby producing smaller and more uniform ZnONPs^38^.

### Zeta potential

Table 1presents the zeta potential values of W-ZnONPs, ZnONPs, and ChNPs. Particles with zeta potential values beyond ± 30 mV are generally considered stable due to strong electrostatic repulsion, which prevents agglomeration^34^. In this study, W-ZnONPs and ZnONPs exhibited zeta potential values of − 25.86 mV and − 28.73 mV, respectively, suggesting moderately stable suspensions.

**Table 1:** Zeta potential values of synthesized nanomaterials.

Nanomaterial	Zeta potential (mV)
W-ZnONPs	–25.86
ZnONPs	–28.73
ChNPs	+ 36.40

The slightly higher negative charge of ZnONPs compared with W-ZnONPs indicates that the phenolic and flavonoid compounds present in *H. sabdariffa *extract effectively functioned as reducing and capping agents, thereby enhancing nanoparticle stability^44^. ChNPs, on the other hand, displayed a high positive charge (+ 36.40 mV), which can be attributed to the extensive deacetylation of chitosan and subsequent protonation of amino groups, conferring multiple cross-linking sites^45^. The electrostatic interaction between negatively charged ZnONPs and positively charged ChNPs suggests strong potential for forming a stable nanocomposite system, which is advantageous for developing multifunctional smart packaging applications.

### DPPH antioxidant activity

**Fig. 6:**
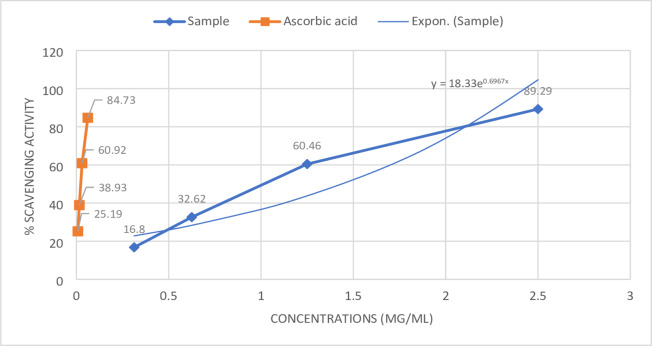
The % Scavenging activity plotted versus the concentrations of the tested samples in mg/mL. Sample refers to SCS.

The radical scavenging activity of the smart composite solution (SCS) was evaluated using the DPPH assay and compared with ascorbic acid as a standard antioxidant. The results are summarized in Table 2 and illustrated in Fig. 6.

**Table 2:** Antioxidant activity results of SCS and ascorbic acid expressed as scavenging activity (%)*.

Sample	Concentration (mg/mL)	% Scavenging activity
SCS	2.5	89.29 ± 3.12^a^
	1.25	60.46 ± 2.69^b^
	0.625	32.62 ± 2.03^c^
	0.313	16.80 ± 1.62^d^
Ascorbic acid	0.062	84.73 ± 4.50^a^
	0.031	60.92 ± 3.73^b^
	0.016	38.93 ± 2.23^c^
	0.008	25.19 ± 2.09^d^

* Values are expressed as mean ± SD (*n* = 3). Different ***superscript*** letters (a–d) within the same column indicate significant differences (*p* < 0.05).

As shown in Fig. 6, the percentage of scavenging activity (%) increased with increasing concentrations of SCS, reflecting enhanced radical scavenging potential. At the highest tested concentration (2.5 mg/mL), SCS achieved 89.29% scavenging activity, which was comparable/surpassing ascorbic acid (84.73% at 0.062 mg/mL). The antioxidant performance declined progressively at lower SCS concentrations (Fig. 6). These results demonstrate that the incorporation of *H. sabdariffa* extract and ZnONPs into ChNPs significantly enhanced the antioxidant capacity of the composite solution. The strong activity can be attributed mainly to the phenolic and flavonoid compounds in *H. sabdariffa*, which are known for their hydrogen-donating and radical-neutralizing properties. ZnONPs may also contribute by generating electron-dense surfaces that facilitate radical quenching. Similar findings have been reported for chitosan-based nanocomposites enriched with bioactive plant extracts and inorganic nanoparticles, which showed improved free radical scavenging activity compared with pure chitosan matrices^35,46–48^. However, the scavenging percentage obtained in this study was comparatively higher, highlighting the synergistic effect of combining anthocyanins with ZnONPs in the nanocomposite system.

### Antibacterial activity

**Fig. 7:**
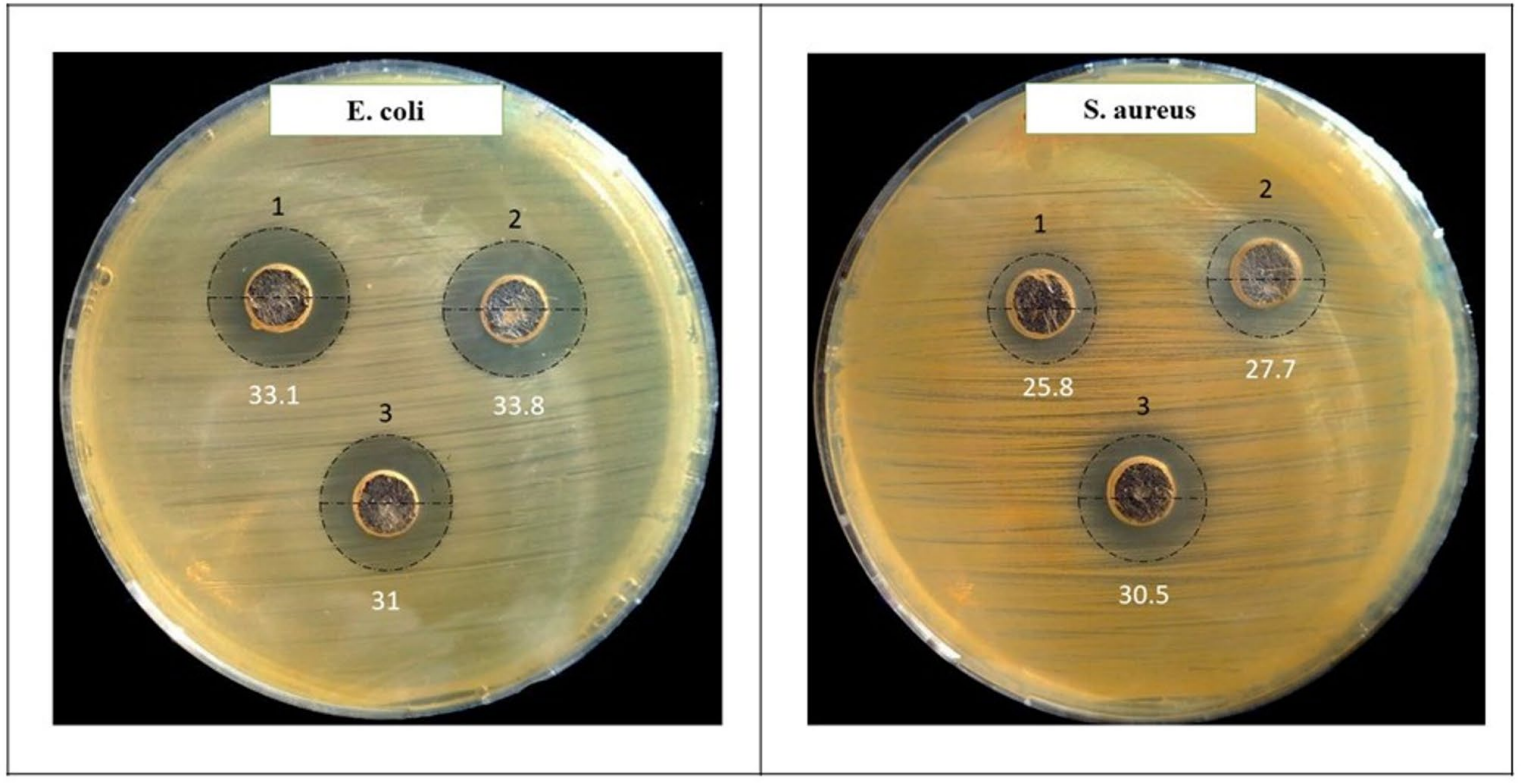
Antibacterial activity of the SCS against *Escherichia coli* and *Staphylococcus aureus*.

As shown in Fig. 7, SCS exhibited antibacterial activity against both *Escherichia coli* and *Staphylococcus aureus*. The interaction between SCS and bacterial cells leads to cellular damage, as the positively charged amino groups (–NH₂) of chitosan interact with negatively charged bacterial surfaces, disrupting the cell membrane^49^. In addition, ZnONPs generate reactive oxygen species (ROS), which induce membrane disintegration and deterioration of cellular proteins and DNA^50^.

The results demonstrated that SCS exerted stronger antibacterial activity against *E. coli* compared to *S. aureus*. This enhanced susceptibility may be attributed to the flavonoid compounds in HE, which can penetrate the inner membrane of Gram-negative bacteria and the cell membrane of Gram-positive bacteria. The antibacterial mechanism against Gram-negative bacteria involves inhibition of DNA gyrase, resulting in apoptosis, whereas for Gram-positive bacteria, the activity mainly depends on membrane disruption, leading to comparatively weaker inhibition^51^. The antibacterial activity of SCS is summarized in Table 3.

**Table 3:** Antibacterial activity of SCS against *Escherichia coli* and *Staphylococcus aureus*.

Microorganisms	1	2	3	Mean ± SD (mm)
*Escherichia coli* (ATCC 10536)	33.1	33.8	31.0	32.63 ± 1.46
*Staphylococcus aureus* (ATCC 6538)	25.8	27.7	30.5	28.00 ± 2.36

### Fourier transform infrared (FT-IR) spectroscopy

**Fig. 8:**
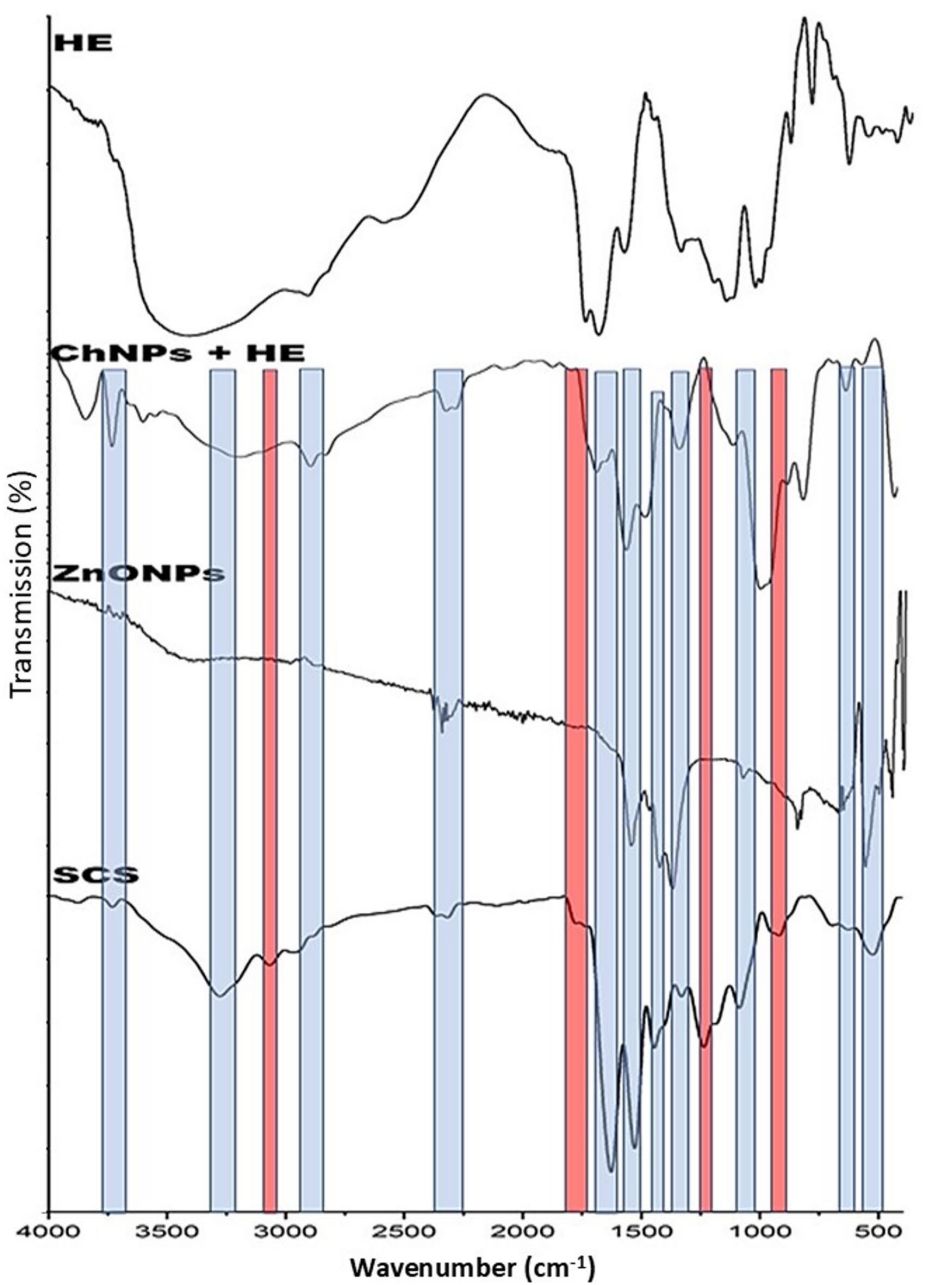
FT-IR spectra of HE, ZnONPs, ChNPs + HE, and SCS. The blue markers indicate peaks originating from ChNPs + HE or ZnONPs, while the red markers highlight new peaks that emerged due to molecular interactions among the components in the SCS. These new absorption bands confirm the successful integration of chitosan, hibiscus extract, and ZnONPs into a nanocomposite structure.

The FT-IR spectral analysis of componenets are blotted in Fig. 8. The FT-IR spectrum of HE showed a broad and intense absorption band at 3422.62 and 2899.48 cm⁻¹, corresponding to O–H stretching vibrations^4^. A distinct peak at 1028.35 cm⁻¹ was assigned to C–O stretching of ester groups^52^,while the peak at 1335.05 cm⁻¹ indicated O–H bending of phenolic compounds^53^. Additional absorption bands at 1734.54 and 1579.90 cm⁻¹ were associated with C = O stretching and aromatic C = C bonds, respectively, whereas the peak at 780.93 cm⁻¹ was attributed to C–H out-of-plane bending^26^. Peaks between 1118 and 1028 cm⁻¹ suggested the presence of anthocyanins, particularly cyanidin-3-O-sambubioside and delphinidin-3-O-sambubioside^54^. The FT-IR spectrum of ChNPs + HE displayed noticeable peak shifts compared with HE alone, reflecting hydrogen bonding and molecular interactions between HE phytocompounds and chitosan functional groups, confirming successful conjugation. The FT-IR spectrum of ZnONPs revealed characteristic peaks at 484.54 and 432.97 cm⁻¹, which correspond to Zn–O stretching vibrations^55^. A peak at 858.25 cm⁻¹ was attributed to tetrahedral coordination of zinc, while the band at 662.37 cm⁻¹ was assigned to Zn–O–Zn stretching modes^56^. Metal–O group vibrations were further confirmed in the region 400–600 cm⁻¹^57^.In addition, absorption peaks at 1543.81 and 956.19 cm⁻¹ were related to C = O and C = C stretching of hibiscus phytocompounds, respectively^58–60^. The peak at 1358.25 cm⁻¹ indicated the presence of phenolic groups derived from hibiscus extract. These results suggest that hibiscus phytochemicals played a dual role as reducing and capping agents during ZnONPs biosynthesis, thereby stabilizing the nanoparticles^61,62^.

The FT-IR spectrum of the SCS nanocomposite exhibited a combination of peaks characteristic of both ChNPs + HE and ZnONPs. Newly emerged peaks (highlighted in red) suggested novel molecular interactions, confirming the successful integration of chitosan, HE phytocompounds, and ZnONPs into a stable nanocomposite matrix.

### Scanning electron microscope (SEM)

**Fig. 9:**
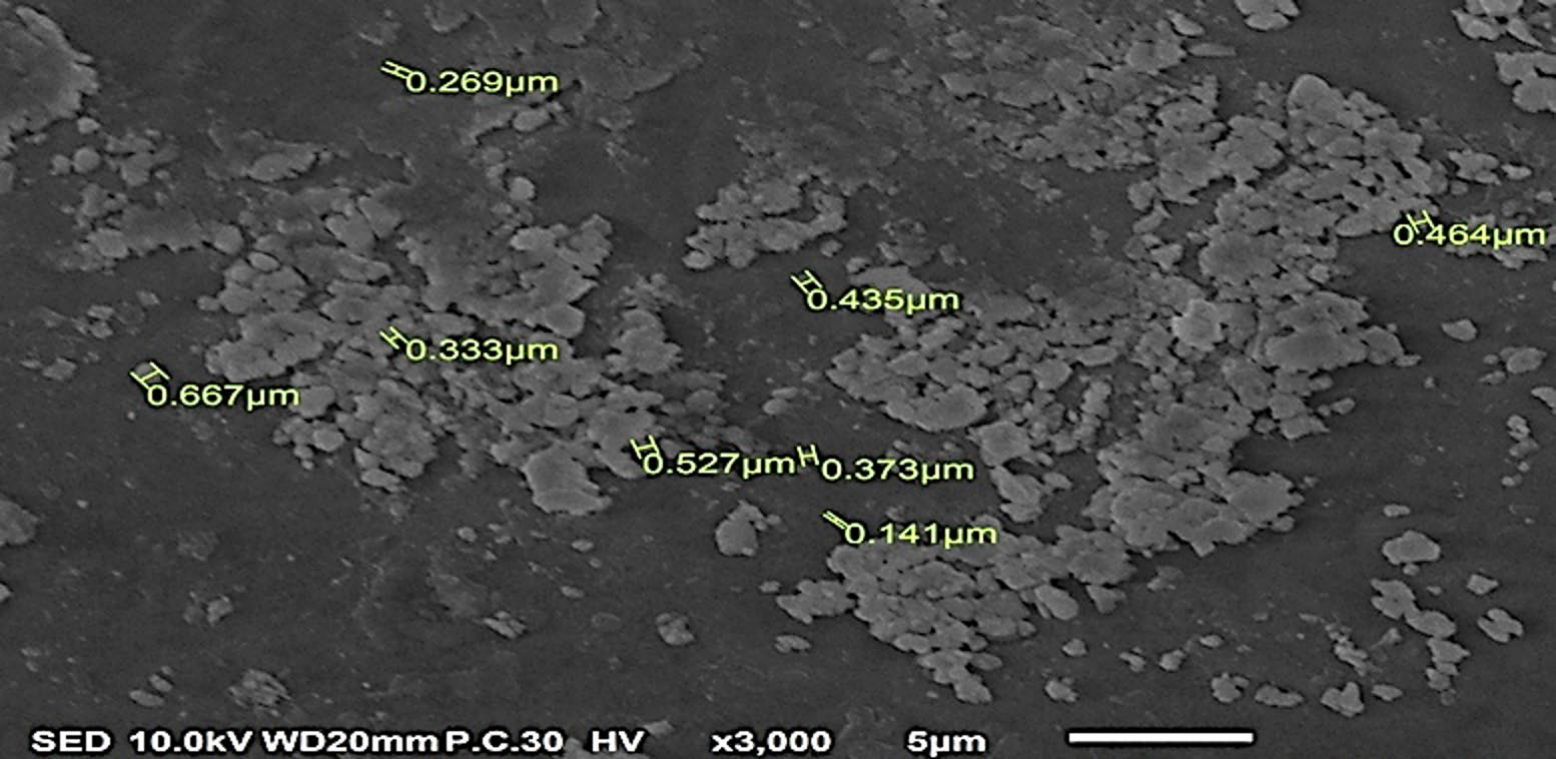
SEM micrographs of the smart chitosan-based system (SCS).

SEM analysis was carried out to investigate the surface morphology and microstructural features of the developed smart chitosan-based system (SCS). As illustrated in Fig. 9, the SEM images revealed that chitosan nanoparticles (ChNPs) were successfully synthesized using the ionic gelation method, with particle sizes ranging from 0.141 μm to 0.667 μm, confirming their nanoscale characteristics. The particles exhibited a heterogeneous morphology, with both spherical and irregular shapes visible. Such variation in morphology is typical for ionically cross-linked chitosan systems, as the degree of deacetylation and protonation of amino groups significantly influences the shape and stability of nanoparticles^63^. The SEM micrographs further demonstrated that zinc oxide nanoparticles (ZnONPs) were successfully incorporated and coated with ChNPs, leading to the formation of a hybrid nanocomposite structure. Although some agglomeration was observed, the nanoparticles maintained a relatively uniform distribution within the chitosan matrix. This agglomeration can be attributed to the high surface energy of ZnONPs and their tendency to form clusters during synthesis and drying processes^64^. Importantly, the encapsulation of ZnONPs within the chitosan network appeared to mitigate excessive clustering, thus improving their dispersibility and enhancing the stability of the final nanocomposite. The hybrid morphology observed is of great significance for the intended application of SCS as an active packaging and preservation system for Nile perch. The spherical nanoparticles provide a high surface area-to-volume ratio, which is essential for effective antibacterial and antioxidant action, while the irregularly shaped particles may enhance barrier properties by creating tortuous diffusion pathways for oxygen and moisture^65^. Furthermore, the uniform distribution of ZnONPs across the chitosan surface ensures their effective contact with microbial cells, maximizing antibacterial activity through reactive oxygen species (ROS) generation and disruption of cell membranes. Compared with previous reports, the morphological features of the present SCS align well with findings from similar studies involving chitosan–metal oxide nanocomposites. For example, **Aouadi et al.**^66^,observed that chitosan–ZnO systems consistently displayed mixed spherical and irregular structures, with nanoparticle agglomeration linked to electrostatic interactions between positively charged chitosan and negatively charged ZnO particles. Likewise, it was reported that bioactive extract-mediated ZnONPs, when embedded in a polysaccharide carrier, maintained uniform distribution despite moderate aggregation^39,48^. These similarities confirm the reproducibility and robustness of the ionic gelation approach employed in the current study. The observed surface features also support the functional properties reported for SCS in earlier sections. The roughened and irregular morphology increases the surface area available for interaction with free radicals, which explains the enhanced antioxidant performance measured via the DPPH assay. Moreover, the presence of ZnONPs embedded across the chitosan surface strengthens the antibacterial effect against both Gram-positive and Gram-negative bacteria, as discussed in Sect. 3.7. SEM therefore provides direct morphological evidence correlating with the improved bioactivity of SCS.

In summary, SEM analysis confirmed the successful formation of a nanocomposite system comprising ChNPs, ZnONPs, and hibiscus extract, with characteristic nanoscale features, moderate agglomeration, and heterogeneous morphology. The microstructural features observed are consistent with those reported in recent literature and support the multifunctional role of SCS in quality monitoring and shelf-life extension of Nile perch.

Micrographs reveal nanosized ChNPs (0.141–0.667 μm) with ZnONPs uniformly distributed across the matrix. Spherical and irregular morphologies were observed, with moderate agglomeration indicating strong interparticle interactions.

### Application of SCS on fish samples

**Fig. 10:**
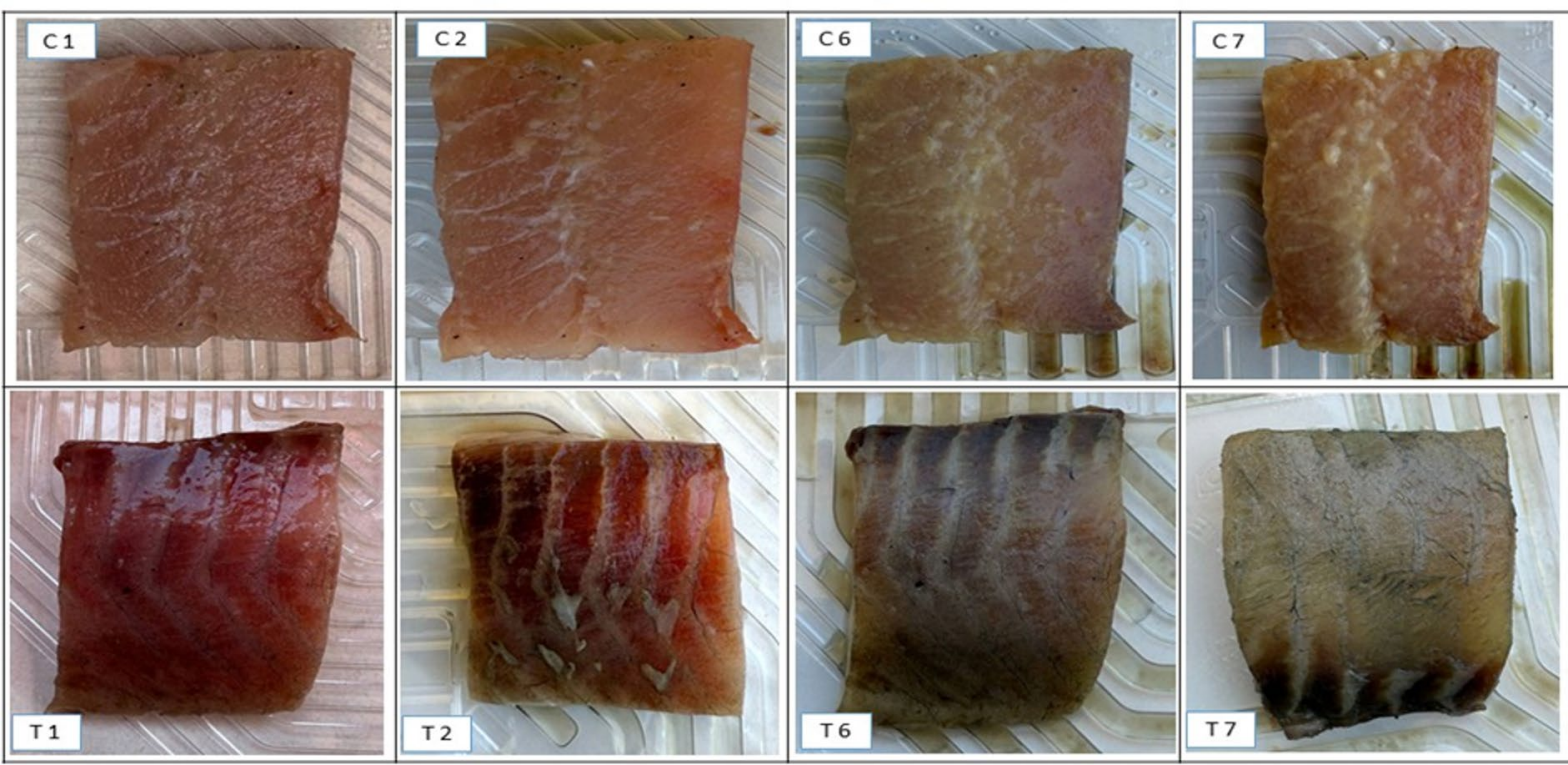
Measurement of color changes and shelf-life status in Nile perch fillets. The figure shows two groups during refrigerated storage at 4 °C for 7 days: the untreated control group (C) and the group treated with smart chitosan solution (SCS) (T). The control fish exhibited rapid spoilage with swelling and off-odor by days 6–7, whereas the SCS-treated fish showed gradual color transitions (red → blue → green) in response to pH changes, indicating delayed spoilage and extended shelf life.

The application of smart chitosan solution (SCS) was evaluated on Nile perch fillets stored under refrigeration (4 °C for 7 days) to determine its efficiency as both a freshness indicator and a preservation tool. As shown in Fig. 10, the untreated control group (C) underwent rapid spoilage by days 6 and 7 (C6, C7), as evidenced by extensive bacterial growth, tissue swelling, and the development of strong off-odors. These findings are consistent with the well-documented perishability of fish, where high water activity, neutral pH, and nutrient-rich muscle promote microbial proliferation and enzymatic breakdown of proteins and lipids^67^.

In contrast, the SCS-treated samples (T) demonstrated markedly different behavior. On day 1 (T1), the fillets presented a distinct red coloration attributed to the anthocyanins present in *Hibiscus sabdariffa* extract. With progression of storage, the fillets shifted from red to blue (T7), and eventually to green at the end of storage, reflecting anthocyanin’s pH-sensitive behavior. This progressive shift is the result of biochemical and microbial spoilage reactions in the fish matrix, which alter surface pH and trigger anthocyanin structural transformations^68^. The observed color transitions therefore provided a visual freshness indicator, allowing for real-time monitoring of spoilage without destructive testing. Notably, the SCS-treated fillets showed only minor swelling by day 7, with no off-odors detected during the entire storage period. This highlights the dual role of SCS as an intelligent spoilage sensor and an active antimicrobial agent. The antimicrobial effect of SCS arises from the combined activity of its components. Chitosan exerts bacteriostatic effects through electrostatic interactions between its positively charged amino groups and the negatively charged bacterial cell walls, leading to leakage of intracellular constituents. ZnONPs contribute by generating reactive oxygen species (ROS) that damage membranes, proteins, and nucleic acids^69^.

In addition, hibiscus-derived flavonoids penetrate bacterial membranes and inhibit DNA gyrase in Gram-negative bacteria or disrupt cell membranes in Gram-positive bacteria, thereby enhancing antimicrobial efficiency^5,51^. The present findings are in agreement with recent studies using anthocyanin-based chitosan films and coatings for monitoring fish spoilage. A study reported that hibiscus anthocyanin-loaded chitosan films exhibited visible colorimetric changes during fish storage, confirming the potential of anthocyanins as freshness indicators^70^. Similarly, others demonstrated that chitosan coatings incorporating natural anthocyanins extended salmon shelf life while providing pH-responsive color changes^71^. However, unlike solid films, the dipping approach employed here ensured uniform coating of the fish surface, enhancing antimicrobial contact and maximizing antioxidant protection. This offers an additional advantage over film-based indicators, which may not cover all food surfaces evenly. Overall, the results demonstrate that SCS could delay spoilage, preserved sensory quality, and provided an easily interpretable freshness indicator in Nile perch during chilled storage. The dipping solution strategy suggests a practical and low-cost approach to intelligent food preservation, combining both bioactive functionality and consumer-friendly freshness monitoring. These outcomes highlight the potential of SCS for application in seafood and other highly perishable foods, representing an innovative advance in smart food packaging technologies.

The pH values of fish fillets showed notable variation (Table 4) during 7 days of storage at 4 °C, with differences observed between the control (untreated) group and the group treated with SCS. The SCS-treated group had firstly lower pH of 6.53, likely because SCS is slightly acidic due to its content of HE. On day 2, the pH dropped in both groups, reaching 6.21 (control) and 6.10 (treated). This drop is attributed to the accumulation of lactic acid from continued post-mortem glycolysis. Such a decrease to pH 6.0–6.5 (or even lower) is typical during the rigor mortis phase. After glycolysis ceases, while pH remains low, the muscle begins to soften again as resolution of rigor occurs. By day 6, the control group reached a pH of 7.23 which is a high value indicating complete spoilage. This rise results from microbial and enzymatic breakdown of proteins, releasing alkaline compounds such as ammonia and volatile bases. A pH exceeding 7.0 is widely considered a strong indicator of advanced spoilage^2,9,53^. In contrast, the SCS-treated group maintained a pH of 6.56 on day 6, demonstrating that SCS effectively controlled pH elevation and extended shelf life.

**Table 4:** Spoilage parameters (pH and TVB-N) of Nile perch fillet coated with SCS during storage at 4 °C for 7 days, compared to control samples.

Storage Day	pH		TVB-*N* (mg/100 g)	
	Control	Treated	Control	Treated
0	6.82 ± 0.38	6.53 ± 0.41	14.52 ± 1.34	12.26 ± 0.92
2	6.21 ± 0.44	6.10 ± 0.52	16.35 ± 2.05	13.67 ± 1.64
6	7.23 ± 0.35	6.57 ± 0.39	28.46 ± 23.21	17.98 ± 1.96
7	7.85 ± 0.42	6.71 ± 0.32	37.65 ± 5.58	18.42 ± 2.09

The limits of TVB-N for fish fillet acceptance was designated as 35 mg/100 g^67^,resulted from breakdown of proteinous compounds by bacterial actions or autolysis enzymes^54,67^. As accessible from Table 4, TVB-N levels of control group were significantly elevated when compared with SCS treatments; onward till the investigation period end. The values remained below the unfit limit up to the 7th day for treated group, whereas the concentration of TVB-N levels in control group exceeded the acceptable levels in the storage end. However, those results suggest that SCS coating can play significant roles in deceleration TVB-N rates and keep fish fillets close to fresh estimates^1,26,54,67^.

A key finding of this work is that the SCS not only delayed microbial spoilage but also provided a clear visual indicator of freshness loss. The transition from red to purple, blue, and ultimately green during refrigerated storage corresponded with spoilage-related pH shifts. This functionality addresses a major consumer need for real-time freshness monitoring, reducing reliance on ambiguous “best before” dates and helping prevent food waste. In addition, the high antioxidant activity (up to 89.29% DPPH scavenging at 2.5 mg/mL) demonstrated the potential of SCS to mitigate oxidative processes, which is crucial in preserving fish muscle proteins and lipids during storage.

The innovation of this research lies in the integration of three functionalities within a simple dipping solution. While most previous studies have focused on smart films or packaging materials, dipping solutions offer several advantages, including uniform application to irregular food surfaces, adaptability to small- and large-scale processing, and low production cost^32,72^. Furthermore, the combination of chitosan with HE-derived anthocyanins and ZnO nanoparticles created synergistic effects that enhanced antimicrobial and antioxidant performance beyond that of individual components. This approach provides a green, natural, and consumer-friendly technology, aligned with the current demand for sustainable food preservation strategies^33,43,50^. Looking ahead, several future perspectives arise from this work. First, comprehensive toxicological assessments are needed to evaluate the safety of long-term exposure to ZnO nanoparticles in real food systems, ensuring compliance with regulatory standards^37,48^. Second, optimization of dipping protocols—such as immersion time, concentration, and reusability of the solution—would support industrial scale-up. Third, further validation across different fish species and other perishable foods could expand its commercial applicability. Additionally, combining SCS with intelligent packaging technologies, such as QR-coded freshness labels or biosensors, may enhance consumer engagement and food traceability^27,68,72^. Finally, consumer perception and acceptance studies should be undertaken, as the visible color change during storage may positively influence purchasing decisions by providing transparency and reassurance of product safety.

## Conclusion

The present study demonstrated the successful development of a multifunctional smart dipping solution (SCS) composed of chitosan nanoparticles, *Hibiscus sabdariffa* extract (HE), and green-synthesized ZnO nanoparticles. The system effectively combined smart, active, and bioactive functionalities, thereby providing a unique approach for both monitoring and extending the shelf life of Nile perch fillets under refrigerated storage. The HE component provided anthocyanins that exhibited pH-dependent colorimetric responses, functioning as a visual indicator of freshness deterioration. ZnO nanoparticles offered strong antimicrobial activity through the generation of reactive oxygen species, while chitosan contributed both antimicrobial and antioxidant activity via its cationic charge and bioactive sites. Together, these components enhanced the quality and safety of fish fillets by reducing microbial growth, delaying off-odor development, and visibly signaling spoilage progression.

In conclusion, the developed SCS represents a novel, multifunctional food preservation system capable of extending the shelf life of Nile perch fillets while simultaneously providing a natural colorimetric signal of freshness deterioration. Its integration of smart, active, and bioactive properties distinguishes it from conventional preservation methods and positions it as a promising tool in modern food biotechnology. By addressing both safety and transparency, this innovation contributes to reducing food waste, improving consumer trust, and advancing sustainable solutions for the seafood industry. Successful extraction of anthocyanin from *H. sabdariffa* flower that showed color variation in different pH levels and preparation ZnONPs using HE. FT-IR, TEM, UV and XRD investigations assure formation of ZnONPs. A smart, active and bioactive dipping solution (SCS) was successfully prepared using HE and ZnONPs loaded on ChNPs. SEM investigation of SCS shows successful formation of nanocomposite of ZnONPs coated with ChNPs. The components of SCS show unique antioxidant and antibacterial activity. The presence of anthocyanin in HE qualifies SCS to change color in different pH values, making it suitable for monitoring the quality of fish and delay spoilage.

From the potential limitations and prospective work, it is encouraged to perform a direct comparison of the functional performance of control SCS system prepared with W-ZnONPs and under identical conditions (e.g. antibacterial activity, antioxidant capacity, or preservation efficacy).

## Data Availability

The data presented in this study are available on request from the corresponding author.
